# Investigating the phytotherapeutic efficacy of *Acacia nilotica* pod ethanolic extract in modulating lipid profile, oxidative stress and inflammation in diet-induced hypercholesterolemia: *in vivo* and *in silico* insights

**DOI:** 10.3389/fphar.2026.1831586

**Published:** 2026-05-07

**Authors:** Khurram Afzal, Hamna Khalid, Asad Abbas, Ralf Weiskirchen, Nudrat Khursheed, Bipindra Pandey, Hassan Raza, Abdul Malik, Tanmoy Dutta, Hina Javed, Javaria Saeed

**Affiliations:** 1 Department of Human Nutrition, Faculty of Food Science and Nutrition, Bahauddin Zakariya University, Multan, Pakistan; 2 Institute of Molecular Pathobiochemistry, Experimental Gene Therapy and Clinical Chemistry (IFMPEGKC), RWTH University Hospital Aachen, Aachen, Germany; 3 Department of Pharmacy, Madan Bhandari Academy of Health Sciences, Hetauda, Bagmati, Nepal; 4 Department of Pharmaceutics, College of Pharmacy, King Saud University, Riyadh, Saudi Arabia; 5 Department of Chemistry, JIS College of Engineering, Kalyani, West Bengal, India; 6 Department of Pharmaceutics, Bahauddin Zakariya University, Multan, Pakistan; 7 Department of Pharmacy, University of Southern Punjab, Multan, Pakistan

**Keywords:** *A. nilotica*, antioxidant activity, cardiac enzymes, high-fat diet-induced hypercholesterolemia, hypolipidemic potential, *in vivo* study, molecular dynamics, oxidative stress markers

## Abstract

**Objective:**

This study aimed to investigate the antioxidant, antihyperlipidemic, antihypercholesterolemic, and hepatoprotective properties of *Acacia nilotica* (*A. nilotica*) pod extracts in male albino Wistar rats.

**Methods:**

*A. nilotica* pod extracts (ANPE) were prepared using ethanol, methanol, acetone, and distilled water. Antioxidant properties were evaluated by determining total phenolic content (TPC), ferric reducing antioxidant power (FRAP) and DPPH (2,2-diphenyl-1-picrylhydrazyl) activity. ANPE were administered at doses of 250, 500, and 750 mg/kg/day to rats with hypercholesterolemia induced by a high-fat diet. Lipid profile, cardiac enzymes (CK-MB and Troponin-I) and oxidative stress markers were measured. In addition, virtual screening and molecular dynamic simulations were performed to assess the potential of *A. nilotica* pod constituents to inhibit oxidosqualene cyclase (OSC), a key enzyme in cholesterol biosynthesis.

**Results:**

The ethanolic extract of *A. nilotica* pods displayed the highest antioxidant activity with a TPC of 61.83 ± 2.60 mg GAE/g, FRAP of 1712.9 ± 4.11 µg Fe/g and DPPH inhibition of 79.47% ± 0.66%. The highest dose (750 mg/kg) of ANPE significantly decreased serum cholesterol and LDL levels, while improving HDL and HDL/LDL ratio (p < 0.05). This dose also improved oxidative stress markers, cardiac enzymes, the atherogenic index, and the Castelli index compared with the hypercholesterolemic control group (p < 0.05). Histopathological examination of liver and kidney tissues confirmed that the highest dose of APNE exerted marked hepatoprotective and renoprotective effects (p < 0.05). *In silico* analyses highlighted a plausible molecular mechanism whereby β-sitosterol of *A. nilotica* interacts with the OSC active site, supporting a hypothesis for the observed reduction in cholesterol biosynthesis.

**Conclusion:**

ANPE exhibits strong antihypercholesterolemic, antioxidant, hepatoprotective, and nephroprotective effects in a rat model of diet-induced hypercholesterolemia. The long-term safety and efficacy of its bioactive constituents should be evaluated in future preclinical and clinical studies. Computational modelling suggests that β-sitosterol from *A. nilotica* may act as a putative OSC inhibitor, providing a mechanistic framework that warrants further *in vitro* and biochemical validation.

## 1 Introduction

Plant foods are consumed worldwide because they are highly nutritious and provide abundant energy content. The growing use of plant-based foods is partly driven by the increasing prevalence of chronic diseases associated with diets rich in animal products. Recommendations for healthy eating state that a person should consume at least 400 g of plant-based foods, including vegetables and fruits, each day (Samtiya et al., 2021). In recent years, medicinal plants have gained significant attention in modern medicine because of their accessibility, affordability, widespread acceptance, and safety, making herbal remedies highly valued globally. Consequently, ensuring the quality, efficacy, and safety of medicinal plants has become a critical concern for both developed and developing nations (El-Saadony et al., 2025). According to the World Health Organization (WHO), traditional medicine originating from medicinal plants benefits 80% of the world’s underprivileged population (World Health Organization, 2024). Additionally, the WHO has documented more than 20,000 species of medicinal plants in its database and recognized them as a potential source of novel pharmaceuticals. Of the bioactive compounds isolated from plants, 74% have been identified through ethnomedicinal practices. Furthermore, it is estimated that 14%–28% of higher plant species exhibit medicinal properties (Panda et al., 2025; Shamim et al., 2025).

*A. nilotica* was first described by Linnaeus in 1773. The genus *Acacia* is one of the largest family of Fabaceae, comprising approximately 1,350 species. *A. nilotica* is also rich in nutrients and has high therapeutic value, with reported potential to prevent, reduce the severity, and treat many infections and detrimental diseases (Saeedi et al., 2020). The parts of the plant employed by Native Americans include roots and rhizomes, flowers, leaves, fruits, seeds, and oils. Physicians have applied these medicaments as powders, pills, suppositories, creams, pastes, and ointments (Omara et al., 2012). Numerous bioactive substances present in *A. nilotica*, such as alkaloids, flavonoids, terpenoids, phenolics, and essential oils, possess pharmacological properties including antibacterial, antioxidant, anti-inflammatory, anticancer, and immunomodulatory activities, which have been extensively investigated. In addition to their great therapeutic potential, bioactive substances from medicinal plants are often preferred because of their natural origin, which is perceived as safer and more environmentally friendly than synthetic drugs (Dar et al., 2023). AORA Health’s market report on the pharmaceutical market suggests that the nutraceutical market is projected to grow by about 50% between 2022–2028, with an average annual growth rate of 8.6% (Pérez-Martínez et al., 2023).

Pods are rich in mucilage, tannins, and saponins (Khaled and Irani, 2021). Phytochemical studies have shown the presence of carbohydrates, proteins, phenols, tannins, saponins, starch, flavonoids, and steroids in the pods of *A. nilotica*. These pods mainly contain 12%–19% tannin, with the amount increasing up to 19%–27% in deseeded pods. Crude protein (15.8%) is also present in dry pods. ANPE are used ethnomedicinally for postpartum wound healing. Decoctions of plant pods have been found to be beneficial in the treatment of urogenital diseases, sexual diseases such as spermatorrhea, loss of viscosity of semen, frequent night discharge, and prevention of premature ejaculation in males (Mansi et al., 2021). Seeds, barks, and leaves have shown antiphlogistic, emetic, diuretic, cathartic, and expectorant properties against cough, fever, hemorrhoids, menstrual complaints, and cancer (Ahmed et al., 2024). Various parts of the plant, including the bark, gum, leaves, flowers, and roots, serve medical purposes for dysentery, diabetes, wound healing, astringency, diarrhea treatment, and toothbrush use (Jaiswal et al., 2023). The yearly productivity of mature trees ranges from 14 to 3,150 pods or approximately 832 pods per tree (Zahra et al., 2020). The main polyphenols in *A. nilotica are* condensed tannin and phlobatannin polyphenols, with minor levels of gallic acid, ellagic acid, catechins, and epigallocatechin-7-gallate (Jain et al., 2025). Plant parts such as roots, leaves, pods, and bark exhibit the greatest amount of tannin and phenolic compounds, which contain specific components like gallic acid, dicatechin, quercetin, robidandiol, β-amyrin, hentriacontan, betulin, sitosterol, kaempferol-3-chlorogenic acid, and glucoside isoquercetin (Batiha et al., 2022).

*A. nilotica* is often regarded as a famine food due to its high nutrient content. Its tender green pods are commonly consumed as vegetables, while the mature seeds are fried or roasted before consumption. Additionally, the people of Rajasthan prepare flour from matured seeds, often blending it with millet flour to create composite flour for traditional dishes, including Indian sweets such as laddu and panjiri (Tiwari et al., 2023). Incorporating *A. nilotica* seed extract at a dosage of 150 mg has been shown to extent shelf life of chicken patties up to 15 days under refrigerated conditions by reducing lipid oxidation. *A. nilotica* seeds, also known as wattle seeds, have been combined with chocolate powder in various ways to create chocolate beverages. *A.* cultivars have been used in conjunction with flaxseeds to make gluten-free bread (Kumari and Swer, 2025).

Cholesterol metabolism is linked to oxidative stress and inflammation (Cardoso and Perucha, 2021). Pro-inflammatory signaling can be triggered and reactive oxygen species (ROS) produced by excess cholesterol or its metabolic byproducts (Guo et al., 2024). Recent reviews highlight that cholesterol overload induces severe redox imbalance and heightens inflammatory pathways in cells. Thus, dysregulated sterol synthesis leads to more ROS and inflammation. Breaking this cycle by inhibiting upstream enzymes can have antioxidant and anti-inflammatory benefits. Cholesterol-lowering interventions broadly improve redox and inflammatory markers. For example, statin therapy (HMG-CoA reductase inhibition) not only lowers cholesterol but also reduces systemic oxidative DNA damage and chronic inflammation (Sørensen et al., 2019). One critical rate-limiting step in the cholesterol biosynthesis route is the cyclization of 2,3-oxidosqualene to lanosterol, catalyzed by oxidosqualene cyclase (OSC, also called lanosterol synthase) (Thoma et al., 2004). OSC was selected as a computational target to explore a theoretical molecular rationale for the efficacy of *A. nilotica* pod extract (Agravat et al., 2025). An OSC inhibitor that reduces cholesterol *in vivo* was used to solve the PDB structure 1W6J (human OSC bound to inhibitor Ro 48-8071), highlighting the druggability of OSC in metabolic diseases. Molecular docking is a computational technique designed to predict the preferred binding orientation (pose) of a small molecule (ligand) when it forms a complex with a macromolecular target (receptor) (Pandey et al., 2024a; Pandey et al., 2024b). Before engaging in extensive and expensive *in vivo* experiments, this prediction power is essential for clarifying structure-activity correlations (SAR). Molecular dynamics (MD) simulations serve as a rigorous validation step for docking results. By analyzing parameters such as root mean square deviation (RMSD) and root mean square fluctuation (RMSF), researchers can determine if a predicted pose is a true energy minimum or a transient artifact (Pandey et al., 2024a; Pandey et al., 2024b). This is especially important for membrane proteins like OSC, where the lipid milieu around the binding site affects its flexibility and accessibility. Based on existing compound-profiling data for *A. nilotica* pods, selected pod constituents were used as ligands in an *in silico* study against the OSC protein receptor to understand the efficacy of specific compounds related to oxidative stress and inflammation.

Despite the growing interest in the phytotherapeutic potential of *A. nilotica*, most studies have focused on its general medicinal properties, with limited research specifically addressing its effects on lipid metabolism, oxidative stress, and inflammation in hypercholesterolemia. While some studies have explored the effects of individual bioactive components, comprehensive investigations of the effects of ANPE in diet-induced hypercholesterolemia remain scarce. In addition, minimal literature has investigated the capability of ANPE to alter oxidative stress and inflammatory signatures, which are the main causes of cardiovascular complications. This study is the first to simultaneously assess the nutritional composition together with biochemical and oxidative stress markers and computational insights into cholesterol metabolism via OSC inhibition with β-sitosterol.

## 2 Materials and methods

### 2.1 Materials

*A. nilotica* (kikar) pods were collected in bulk from Pansar market in Multan, Pakistan, for research purposes and were deposited in the Herbarium of the Faculty of Food Science and Nutrition at Bahauddin Zakariya University in Multan, Pakistan (MSHND-2025-34). Egg yolk and lard powder were purchased from the local market in Multan, while cholesterol, pig bile salt and paraformaldehyde tissue fixative were procured from the Pharmaceutical Market in Multan.

The standard basal diet consisted of corn starch (68%), casein (11%), wheat bran (7%), cellulose (5%), soybean oil (4%), minerals (2%), and vitamins (1%). The high-fat diet was composed of 60.3% standard basal diet, sucrose (15%), egg yolk powder (10%), lard (12%) cholesterol (1.5%), pig bile (1%) and salt (0.2%), prepared according to the formula and dried at 45 °C.

The chemicals used in the study included DPPH (Merck, Darmstadt, Germany). Sodium carbonate (Thermo Fisher Scientific, United States), gallic acid (Thermo Fisher Scientifc), acetone (Thermo Fisher Scientific), ethanol (Thermo Fisher Scientific, India), methanol (Thermo Fisher Scientific), ethyl acetate (VWR International, India), and Hematoxylin and Eosin (Abcam, United Kingdom). All chemicals and reagents used in this study were of analytical grade.

### 2.2 Instruments

The following instruments were used: UV-1800 Spectrophotometer (Shimadzu Corporation, Kyoto, Japan), iMARK Microplate Reader (Bio-Rad Laboratories, Hercules, CA, United States), Axio Observer Z1 Microscope (Carl Zeiss AG, Oberkochen, Germany), R-300 Rotary Evaporator (Buchi Labortechnik AG, Flawil, Switzerland), 5,804 centrifuge (Eppendorf AG, Hamburg, Germany), Adventurer Pro AV114 Weighing Scale (Ohaus Corporation, Parsippany, New Jersey, United States), and FA-200 Analytical Balance (A&D Company Ltd., Tokyo, Japan). *In silico* analysis was conducted using AutoDock Vina (The Scripps Research Institute, United States) for molecular docking, GROMACS (University of Groningen, Netherlands) for molecular dynamics simulations, and bioinformatics databases such as PubChem, PDB, and UniProt (United States, United Kingdom). Statistical data analysis was carried out using IBM SPSS Statistics (Version 26. o, IBM Corp., Armonk, New York, United States).

### 2.3 Extract preparation

Dried *A. nilotica* pods were ground into a coarse powder. The powdered material (25 g) was macerated in 100 mL of 70% ethanol (v/v) (Sigma-Aldrich, United States) and 30% distilled water at room temperature, protected from light for 72 h with occasional stirring. The mixture was then filtered, and the filtrate was concentrated to dryness using a rotary evaporator (Model No: BK-RE-1A, BIOBASE, Jinan, China) at 40 °C–50 °C. The resulting crude *A. niloica* pod ethanolic extract (ANPE) was stored in an airtight container at 4 °C until further use (Amira et al., 2020).

### 2.4 Total phenolic content

Total phenolic content (TPC) was determined using the Folin-Ciocalteu method, following established protocols (Shamim et al., 2025). Specifically, 2 mL of Folin-Ciocalteu reagent was added to a test tube containing 1 mL of the sample extract and 2 mL of 7.5% Na_2_CO_3_ solution (Merck, United States). The mixture was then incubated in the dark for half an hour. Subsequently, the absorbance at 765 nm was measured using a spectrophotometer (Model number: BK-UV1600G, BIOBASE, Jinan, China). The TPC was expressed in milligrams per gram of the extract (mg GAE/g).

### 2.5 Antioxidant analysis

#### 2.5.1 2,2-Diphenyl-1-picrylhydrazyl radical scavenging activity

The ability of the *A. nilotica* extract to scavenge radicals was assessed using the DPPH test. A 0.5 mL sample of the extract combined with a 3 mL aliquot of DPPH solution (0.004% in methanol) (Merck, United States). The absorbance of the solution at 517 nm was measured after 20 min of incubation at 27 ^°^C using a UV Visible Spectrophotometer (Infitek, SP-MUV6000, Jinan, China). The antioxidant capacity was quantified as a percentage of DPPH radical scavenging (Shamim et al., 2025).

#### 2.5.2 Ferric reducing antioxidant power assay

The Ferric Reducing Antioxidant Power Assay was used to assess antioxidant ability by measuring the transition of Fe^3+^-TPTZ complex to the Fe^2+^-TPTZ complex. Incubation of the same extract and FRAP reagent, consisting of acetate buffer, TPTZ, and FeCl_3_ solution (Merck, United States), was carried out at 37 °C for 10 min. Absorbance was measured at 593 nm after incubation. Results were obtained using a standard curve prepared with FeSO_4_ (Merck, United States) (Shamim et al., 2025).

### 2.6 *In vivo* study

#### 2.6.1 Experimental design of animal study

Twenty-five male Wistar-albino rats, each weighing 160 ± 10 g and aged 8 weeks, were obtained from the University of Lahore’s animal facility. These rats were housed in polypropylene cages, with five rats per cage, under controlled conditions: a temperature of 22 °C ± 2 °C, relative humidity between 50% and 60%, and a 12-h light/dark cycle. Throughout the study, the rats had free access to a standard basal diet and water. Before starting the high-fat diet, the rats were kept in well-ventilated cages and fed a standard poultry diet for 1 week. The experimental setup consisted of five groups, each containing five male Wistar-albino rats, treated according to Table 1 following established protocols (Khursheed et al., 2026). The rats were randomly assigned into experimental groups after an acclimatization period through simple random sampling. *A. nilotica* pod extract was administered daily through an oral gavage tube mixed with the standard basal diet during the experiment. The positive control group included normal rats that received a standard diet throughout the study period. The G_2_ group included hypercholesterolemic rats that received standard diet and no therapeutic interventions were given. Groups G_3_ to G_5_ included hypercholesterolemic rats that were treated with different doses of the ANPE extract, administered orally through an oral gavage feeding tube at 250, 500, 750 mg/kg body weight (BW) of the experimental animals. The dose selection of the extract was based on previously reported toxicological studies of *A. nilotica* pods extract (Alli et al., 2015; Abdulhamid et al., 2019; Omara et al., 2012), representing concentrations that are far beyond the reported LD_50_ for both oral and intra-peritoneal routes of administration, which are over 3,800 mg/kg BW as established by others (Baba et al., 2022). The experimental timeline consisted of 8 weeks, during which hypercholesterolemia was induced in the respective groups by feeding a high-fat diet (HFD) for the initial 4 weeks. Following induction, treatment with ANPE extract was initiated and continued for the subsequent 4 weeks (weeks 5–8) as mentioned by (Khursheed et al., 2026).

**TABLE 1:** Experimental design for *in-vivo* study.

Groups	Treatments	Dosing regimen
G_1_	Positive control	SBD
G_2_	Negative control	HFD
G_3_	ANPE (250 mg/kg BW)	HFD supplemented with ANPE 250 mg/kg/day
G_4_	ANPE (500 mg/kg BW)	HFD supplemented with ANPE 500 mg/kg/day
G_5_	ANPE (750 mg/kg BW)	HFD supplemented with ANPE 750 mg/kg/day

Abbreviations used: HFD, High-fat diet; SBD, standard basal diet; BW, body weight; ANPE, *Acacia nilotica* pods ethanolic extract.

#### 2.6.2 Ethical considerations

The study strictly adhered to the ethical guidelines set by the Bioethical Committee of Bahauddin Zakariya University, Multan (Committee number: 129/04-2024). The welfare of the animals was a top priority, and steps were taken to ensure they were treated humanely and not subjected to any suffering. Prior to commencing the experiments, all animals were randomly allocated to various groups. This study also strictly followed the regulations and guidelines set by the National Institutes of Health (NIH). Measures were taken to reduce animal distress in accordance with the principles of the National Center for the Replacement, Refinement, and Reduction of Animal Research (NC3Rs), as outlined in the Animal Research Reporting of *In vivo* Experiments (ARRIVE) guidelines.

#### 2.6.3 Euthanasia of rats and blood sample collection

After the treatment phase, the rats were fasted overnight and anesthetized using a ketamine/xylazine combination (80/10 mg/kg BW). Blood was collected *via* cardiac puncture with sterile syringes, allowed to clot, and then centrifuged at 3,000 rpm for 10 min to separate the serum. The plasma and serum samples were stored at −20 °C for subsequent biochemical, hematological, lipid profile, and cellular enzyme analyses. The animals were euthanized by cervical dislocation, and their livers and kidneys were promptly excised, rinsed with ice-cold saline, weighed, and sectioned for biochemical analysis.

#### 2.6.4 Induction of disease

The experimental animals were acclimatized, and physical and serological experiments were conducted to group them into hypercholesterolemic and non-hypercholesterolemic groups. Rats in the hypercholesterolemic group were given a high-fat diet following established protocols (Yang et al., 2025), while rats in the non-hypercholesterolemic group were fed a standard healthy diet over a period of 6 weeks. After sacrifice, serum lipid profile and liver function analysis were conducted by drawing blood through the tail vein to confirm the induction of disease. All animals underwent daily physical examinations, and their BWs were monitored weekly. Feed intake and water consumption were measured throughout the study period.

### 2.7 Serum biochemical, hematological, lipid profile, and cellular enzyme assay

Blood was collected for biochemical testing on days 0 and 28 of the study. After a 28-day duration, all rats received ketamine (75 mg/kg BW) followed by dissection using a surgical blade No. 24. Blood was obtained via cardiac puncture and collected in anticoagulant EDTA (2%) and gel vials for hematological and biochemical examinations. Subsequently, the serum was separated from the gel tube by centrifuging it through a centrifuge machine (Model: H2050R) at 5,000 rpm for 10 min at 40 °C, and the serum was stored in Eppendorf tubes. Vital organs, such as the liver and kidney, were excised, blotted dry, and weighed to calculate the relative organ indices according to standard procedures (National Research Council, 2011). All biochemical tests were performed under blind conditions to minimize bias, with investigators unaware of group assignments during sample processing and data analysis.

#### 2.7.1 Biochemical examination

Liver and renal functions were assessed using a semiautomatic analyzer. Serum proteins were analyzed using an automatic chemistry analyzer. Cardiac enzymes were analyzed using an automated immunoassay analyzer. Oxidative stress biomarkers were evaluated by determining lipid peroxidation and antioxidant enzyme activities using a UV-Vis spectrophotometer (Shamim et al., 2025; National Research Council, 2011).

#### 2.7.2 Plasma lipid analysis

Plasma lipids were extracted using a chloroform/methanol mixture (2:1, v/v) by following the method described by Sung et al. with slight modifications (Sung et al., 2020). The total lipid content in the plasma extracts was determined through solvent evaporation using a rotary evaporator. Plasma lipid parameters, such as total cholesterol (TC), high-density lipoprotein cholesterol (HDL-C), and triacylglycerol (TG) levels, were calculated using an enzymatic method with commercial kits. Low-density lipoprotein cholesterol (LDL-C) was quantified using the Friedewald equation.

#### 2.7.3 Atherogenic index

The Atherogenic Index (ATH) was calculated using the following formula (Castelli et al., 1992):

$$\text{Atherogenic index }\left(\%\right)=\frac{Total\;cholesterol-HDL\;Cholesterol}{HDL\;Cholesterol}$$

An increase in this index indicates an increased risk of cardiovascular disease, and it is commonly used to assess the risk of atherosclerosis. Total cholesterol and HDL-C were quantified using the enzymatic method, and the results were used to obtain the ATH. This indicator helps identify individuals experiencing an imbalance in their lipid profiles and therefore are at risk for heart disease.

#### 2.7.4 Superoxide dismutase activity assay

The activity of superoxide dismutase (SOD) was measured using an established method (Kumar et al., 2020) with slight modifications. This method enables the spectrophotometric measurement of the absorbance of a colored complex formed during the auto-oxidation of pyrogallol at 412 nm for 3 min at 30-s intervals. One unit of SOD activity was defined as the amount of enzyme required to inhibit auto-oxidation by 50% inhibition per minute.

#### 2.7.5 Catalase activity assay

Catalase activity (CAT) was measured using a method previously described (Hadwan, 2018). The reduction in absorbance due to H_2_O_2_ consumption was observed at 240 nm over a period of 3 min, with readings taken at 30-s intervals. CAT activity was quantified in µmoles of H_2_O_2_ hydrolyzed per minute, calculated using a molar extinction coefficient of 43.6 M^-1^cm^-1^.

#### 2.7.6 Malondialdehyde level assay

Malondialdehyde (MDA) levels were tested using the thiobarbituric acid reactive substances (TBARS) method (Ohkawa et al., 1979). In brief, 1 mL of the sample was mixed with 1 mL of 20% trichloroacetic acid (TCA) and centrifuged at 3,000 rpm for 10 min. Subsequently, 1 mL of 0.67% thiobarbituric acid (TBA) was added to the supernatant and heated to 95 °C for 30 min. The absorbance at 532 nm was measured using a spectrophotometer after cooling. The concentration of MDA was determined based on the absorbance in the presence of the molar extinction coefficient of MDA (1.56 × 10^5^ M^-1^ cm^-1^) and expressed in nmol/mL. Elevated MDA levels suggest the presence of oxidative stress and indicate lipid peroxidation (Tanvir et al., 2026).

### 2.8 Histopathological examination

For histological evaluation, liver and kidney tissues were preserved in formalin fixative. After fixation, the tissues underwent a dehydration process using ethanol solutions of increasing concentrations, followed by clearing with xylene, and then embedding in paraffin. A rotary microtome (Leica RM2235, Leica Microsystems, Deerfield, IL, United States) was used to slice the sections. These sections, each 5 µm thick, were stained with Harris hematoxylin and eosin (H&E) and observed under a bright field microscope (Leica DM500 with ICC50 W CAM, Leica Microsystems). In the liver, the histopathological assessment of tissue damage included factors such as necrotic or damaged hepatocytes, central vein congestion, sinusoidal dilation, inflammatory cell infiltration, and steatosis. Conversely, the kidney evaluation focused on glomerular damage, tubular degeneration, cast formation, and interstitial inflammation. The severity of organ damage was semi-quantitatively scored in the histopathology core facility of Bahauddin Zakariya University using anonymized slides on a 6-point scale (0–5), where 0 indicates normal, 1 is mild, 2 is moderate, 3 is moderate to severe, 4 is severe, and 5 is highly severe, as previously described by researchers (Schafer et al., 2018).

### 2.9 *In silico* study

#### 2.9.1 Molecular docking study

The Protein Data Bank provided the three-dimensional structure of the functionally significant protein linked to oxidosqualene cyclase (PDB access. no.: 1W6J) (http://www.rcsb.org). Modulating OSC directly affects cellular sterol flux and downstream cholesterol-driven processes that underpin oxidative stress and inflammation. Oxidosqualene cyclase catalyzes the committed cyclization of 2,3-oxidosqualene to lanosterol, a crucial upstream step in cholesterol biosynthesis. OSC is a mechanistically appropriate and scientifically justifiable receptor choice because it connects the biochemical and histological data to a tenable molecular mechanism-disturbance of sterol synthesis and lanosterol/cholesterol-mediated redox/inflammatory signaling.

The AutoDock Tools software program was then used to build receptor architectures for molecular docking by removing heteroatoms and adding polar hydrogens and Gasteiger partial charges (Thapa et al., 2025a; Thapa et al., 2025b). The activity of the existing compounds of *A. nilotica* pod*s* was used as ligands for the Molecular Docking study. The details of the ligands are summarized in Table 2. After undergoing geometry optimization, these ligand structures were docked into the binding site using AutoDock Vina (Tiwari et al., 2023), which computed docking scores to measure binding affinity. To describe certain interaction types and binding modes, the resulting binding poses were displayed and examined using PyMOL and Discovery Studio (Batiha et al., 2022; Tiwari et al., 2023).

**TABLE 2:** Selected ligand details for molecular docking study.

Compound name	Solvent source	PubChem CID	Activity	Structure of the ligands	References
Gallic acid	Ethanol/Water	370	Identified as a major phenolic in *A. nilotica* ripe fruit (pod) extracts. Exhibits very strong antioxidant activity), consistent with the high radical-scavenging effects seen for the pod extract	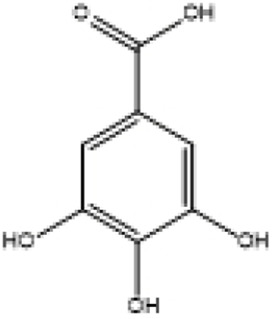	Thapa et al. (2025a), Thapa et al. (2025b), Rawal et al. (2025)
Catechin	Methanol	9,064	Abundant in *A. nilotica* pods and isolated polyphenolic fraction. Exhibits strong antioxidant activity, explaining the extract’s high radical-scavenging capacity and antioxidant effects	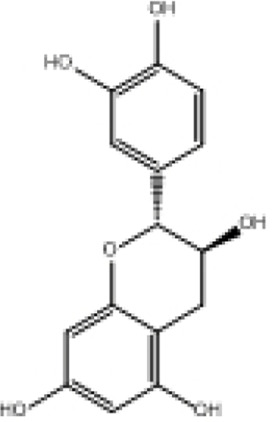	Thapa et al. (2025a), Thapa et al. (2025b)
Epigallocatechin gallate (EGCG)	Methanol	65,064	Found in the pods; noted for its role in reducing systemic inflammation and hepatoprotection	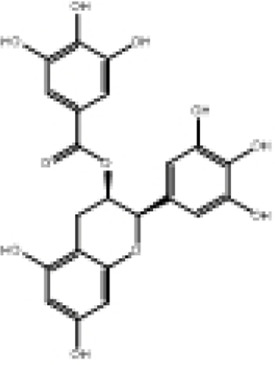	Foyzun et al. (2022)
Ellagic acid	Ethanol	5,281,855	Reported as a significant polyphenolic constituent of the pods, associated with lipid-lowering and antioxidant effects	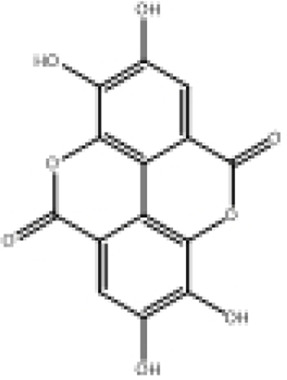	Batiha et al. (2022)
Quercetin	Ethanol	5,280,343	Specifically isolated from pod extracts, a well-known anti-inflammatory flavonoid; contributes to the extract’s inhibition of inflammatory markers. Quercetin has a broad antioxidant/inflammatory efficacy	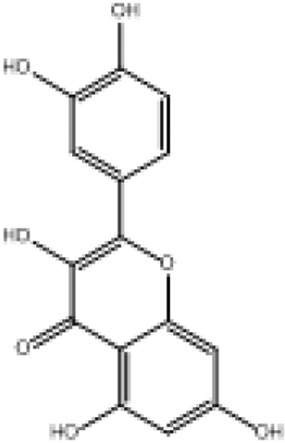	Batiha et al. (2022)
β-Amyrin	Acetone	73,145	A pentacyclic triterpene reported to be present in the pods. Exerts significant anti-inflammatory, antioxidant	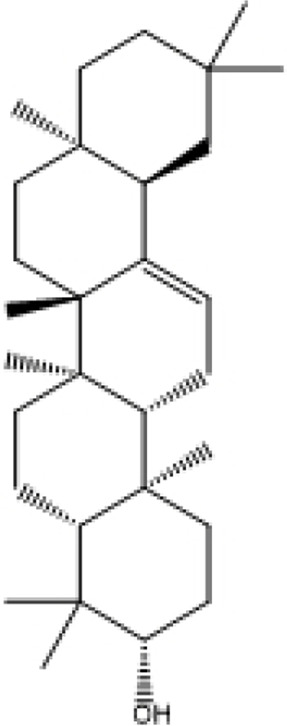	Tiwari et al. (2023)
Betulin	Acetone	72,326	Identified alongside β-amyrin in the pods; a triterpenoid that showed dose-dependent antioxidant. Possesses significant anti-inflammatory activity by preventing the production of pro-inflammatory mediators	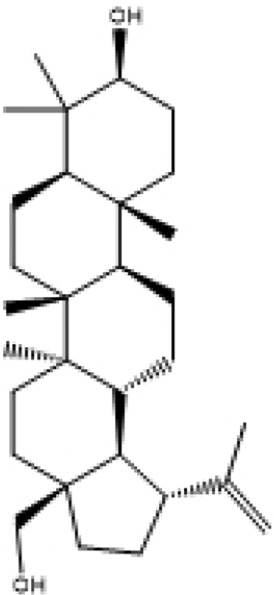	Tiwari et al. (2023)
β-Sitosterol	Acetone	222,284	Confirmed as a sterol constituent present in the pods. Exhibits strong anti-inflammatory, antioxidant effects	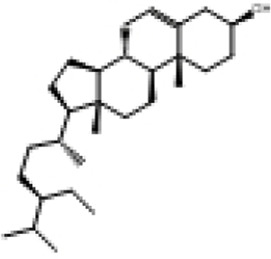	Tiwari et al. (2023), Hafez et al. (2024)
Kaempferol	Ethanol/acetone	5,280,863	Identified in the pods. A flavonol with strong antioxidant capacity AND key antioxidants	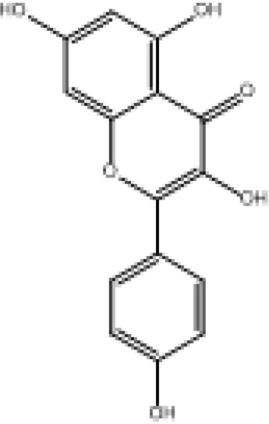	Hafez et al. (2024)
Chlorogenic acid	Ethanol/Water	1,794,427	Specifically mentioned as a glucoside present in the pods. Potent dietary polyphenol with significant antioxidant, anti-inflammatory	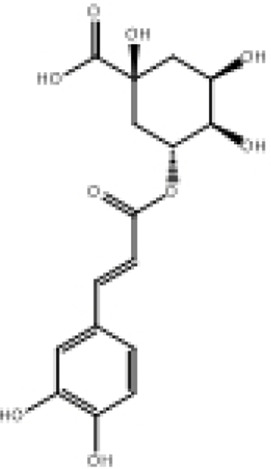	Tiwari et al. (2023)

#### 2.9.2 Molecular dynamics simulations

Molecular dynamics (MD) simulations were conducted for the top ligand based on docking scores with the same protein, OSC, after molecular docking studies. CHARMM-GUI was used to generate all simulated systems, including protein-ligand complexes and apo proteins (Nandi et al., 2024). The protein-ligand complex was immersed in a cubic simulation box containing TIP3P water molecules (Shukla and Tripathi, 2020), ensuring a minimum separation of 10 Å between the solute and the box boundary to avoid artefacts arising from periodic images. Potassium and chloride ions were added to reach an ionic concentration of 0.15 M to mimic physiological conditions. Proteins were parameterized using the CHARMM36 all-atom force field (Jo et al., 2008), while ligands were parameterized using the CGenFF force field (Jorgensen et al., 1983). The systems underwent 1,000 steps of energy minimization using the Adopted Basis Newton–Raphson method prior to the start of the production phase. Equilibration was conducted sequentially with a 1 ns simulation under the NVT ensemble, followed by a 5 ns simulation under the NPT ensemble. Subsequently, 50 ns production molecular dynamics simulations were carried out in GROMACS 2022 as described (Huang and MacKerell, 2013) at 300 K and 1 atm pressure. The Particle Mesh Ewald (PME) technique was used to treat long-range electrostatic interactions (Vanommeslaeghe et al., 2010), with a 12 Å limit used for short-range van der Waals and Coulomb interactions. The simulations were run with a 1.0 fs integration time step, and coordinates were saved every 20 ps for further analysis. Structural stability and dynamic behavior were assessed using standard GROMACS utilities, and binding free energies of the complexes were estimated using the MM-GBSA approach (Abraham et al., 2015).

### 2.10 Data processing

All experimental data were subjected to statistical analysis to determine the level of significance, as described (Shamim et al., 2025) using IBM SPSS Statistics (Version 26.0). Data were analyzed using two-way analysis of variance (ANOVA) followed by Tukey’s HSD *post hoc* test for mean comparison at 5% level of significance (p < 0.05).

## 3 Results

### 3.1 Proximate analysis

Proximate analysis of *A. nilotica* pods revealed the following nutritional components: moisture content of 9.50% ± 0.48%, fat content of 0.12% ± 0.01%, protein content of 6.00% ± 0.30%, crude fiber of 18.40% ± 0.15%, ash content of 6.70% ± 0.34%, and nitrogen-free extract (NFE) of 41.57% ± 0.40%. These results provide detailed insights into the nutritional composition of the pods, which are essential for understanding their potential use in food and health applications (Supplementary Table S1).

### 3.2 Antioxidant activity of *A. nilotica*

As shown in Table 3, TPC, FRAP, and DPPH assays were used to determine the antioxidant activity of *A. nilotica* pod extracts. The lowest overall TPC was in the water extract (47.53 ± 1.19 mg GAE/g) (p < 0.01), while the highest amount was in the ethanol extract (61.83 ± 2.60 mg GAE/g). In the FRAP assay, the highest reducing power (1712.9 ± 4.11 µg Fe/g) was observed in the ethanol extract, followed by the methanol (1,576.5 ± 2.74 µg Fe/g) and water (584.8 ± 1.30 µg Fe/g) extracts (p < 0.01). The highest DPPH radical scavenging activity was seen in the ethanol extract (79.47% ± 0.66%).

**TABLE 3:** Antioxidant properties of pods extract.

Solvent	DPPH (%)	TPC (mg GAE/g)	FRAP (µg Fe/g)
Ethanol	79.47 ± 0.66	61.83 ± 2.60	1712.9 ± 4.11
Acetone	67.97 ± 6.63	56.83 ± 2.18	1,549.0 ± 2.58
Methanol	76.83 ± 5.42	59.23 ± 1.61	1,576.5 ± 2.74
Water	63.33 ± 2.33	47.53 ± 1.19	584.8 ± 1.30

### 3.3 Water intake of experimental animals

**FIGURE 1:**
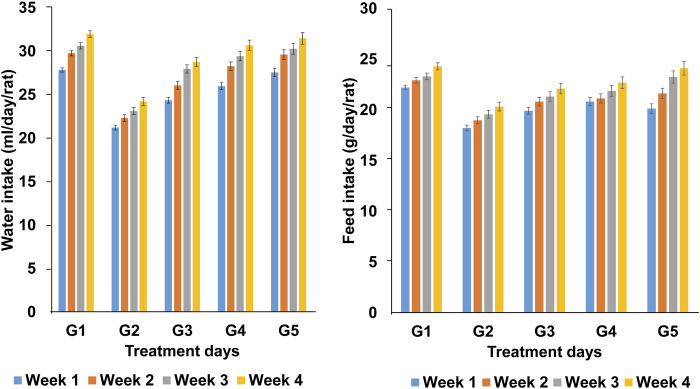
Water intake and feed intake of experimental animals during the study period. Weekly water and feed intake of Wistar rats receiving a standard basal diet (G_1_), high-fat diet control (G_2_), or high-fat diet supplemented with Acacia *nilotica* pod ethanolic extract (ANPE) at 250, 500, or 750 mg/kg body weight per day (G_3_, G_4_, and G_5_, respectively) over the 4-week treatment period following disease induction. The bars represent the mean daily intake per rat for each group in weeks 1-4 of treatment (n = 5 rats per group).

The water intake of the rats during the 4-week trial period of ANPE treatment is shown in Figure 1. The amount of water consumed by all experimental groups varied slightly, with some weeks showing small decreases followed by increases. During the hypercholesterolemia induction period, water intake increased for all groups, with the positive control group consistently consuming less water than the others. Groups treated with *A. nilotica* pod extract exhibited slightly lower water consumption than the positive control group from the beginning of the treatment period. However, the water intake of the treatment groups gradually increased, particularly in the later weeks (weeks 3 and 4). This increase may indicate a growing need for water due to altered physiological needs or an accelerated metabolic rate. In terms of feed intake, the positive control group had the highest feed intake each week, while the negative control group had the lowest. The treatment groups receiving *A. nilotica* pod extract at 250 mg/kg BW/day and 500 mg/kg BW/day showed intermediate feed intake, and the groups treated with 750 mg/kg BW/day had low feed intake, comparable to the negative control group.

### 3.4 Effect of *A. nilotica* pods powder on organ weight on hypercholesterolemic rats

The results of the study provided body and organ weight data that showed significant differences between the various groups. The impact of induced hypercholesterolemia was evident as the BW was highest in the positive control group (G_2_) (220.02 ± 5.08 g). The weight of G_5_ (181.29 ± 5.08 g) was the lowest among all the treated groups (G_3_, G_4_, and G_5_), suggesting the potential impact of *A. nilotica* pod treatment. The liver weight in the G_2_ group (8.12 ± 0.18 g) was the highest, indicating hepatic enlargement associated with hypercholesterolemia. The liver weight (g) of the G5 group showed a significant decrease (7.54 ± 0.18 g), demonstrating a dose-dependent effect of the treatment, despite significant variations in BW (p < 0.05). The highest spleen weight was recorded in G_2_ (0.488 ± 0.014 g), which was significant (p < 0.05) and could indicate activation of the immune system due to hypercholesterolemia. Overall, these findings suggest that the antioxidative and lipid-lowering properties of *A. nilotica* have a moderating influence on body and organ weights, particularly reducing liver size. Further studies are needed to elucidate the mechanisms underlying these effects (Table 4).

**TABLE 4:** Effect of different treatments on body and organ weights in hypercholesterolemic rats.

Parameters	G_1_	G_2_	G_3_	G_4_	G_5_	F-ratio
Body weight (g)	173.35 ± 5.08^d^	220.02 ± 5.08^a^	198.45 ± 5.08^b^	189.24 ± 5.08^bc^	181.29 ± 5.08^cd^	25.06**
Heart weight (g)	0.540 ± 0.017^a^	0.562 ± 0.017^a^	0.560 ± 0.017^a^	0.550 ± 0.017^a^	0.542 ± 0.017^a^	0.72^NS^
Kidney weight (g)	0.452 ± 0.015^a^	0.478 ± 0.015^a^	0.470 ± 0.015^a^	0.460 ± 0.015^a^	0.444 ± 0.015^a^	1.77^NS^
Liver weight (g)	7.89 ± 0.18^ab^	8.12 ± 0.18^a^	7.86 ± 0.18^ab^	7.66 ± 0.18^ab^	7.54 ± 0.18^b^	2.94*
Lung weight (g)	1.60 ± 0.03^a^	1.63 ± 0.03^a^	1.63 ± 0.03^a^	1.62 ± 0.03^a^	1.60 ± 0.03^a^	0.43^NS^
Spleen weight (g)	0.422 ± 0.014^b^	0.488 ± 0.014^a^	0.460 ± 0.014^ab^	0.450 ± 0.014^ab^	0.420 ± 0.014^b^	8.39**
Total organ weight (g)	7.47 ± 0.20^ab^	6.81 ± 0.20^c^	6.89 ± 0.20^bc^	7.29 ± 0.20^abc^	7.51 ± 0.20^a^	5.29**

Means having similar alphabets (a‐d) do not differ significantly (p-values >0.05), ANOVA, test followed by the *post hoc* Tukey’s HSD, test, G_1_; SBD, G_2_; Hypercholesterolemia + SBD, G_3_; Hypercholesterolemia + SBD + ANPE, 250 mg/kg/day, G_4_; Hypercholesterolemia + SBD + ANPE, 500 mg/kg/day, G_5_; Hypercholesterolemia + SBD + ANPE, 750 mg/kg/day; NS, not significant; *p < 0.05; **p < 0.01.

### 3.5 Effect of *A. nilotica* pods powder on hematological evaluation in hypercholesterolemic rats

Hematological analysis revealed that supplementation with *A. nilotica* improved several blood parameters in hypercholesterolemic rats. The erythrocyte sedimentation rate (ESR) was highest in the hypercholesterolemic control group (2.76 mm/h) and decreased in a dose-dependent manner, reaching 1.58 mm/h in G5, indicating reduced inflammation. Hematocrit (HCT) was highest in the normal control (44.52%) and lowest in the positive control group. Hemoglobin (Hb) levels (9.95–11.10 g/dL) remained stable across all groups, confirming no adverse effects on erythropoiesis. The mean corpuscular hemoglobin (MCH) level increased significantly in the treated groups (16.03 pg in G3 and 15.50 pg in G_4_). Red blood cell (RBC) counts were reduced in the hypercholesterolemic group (6.72 ×10^6^/µL) and improved with treatment (7.14 ×10^6^/µL in G5) (Supplementary Table S2).

### 3.6 Effect of *A. nilotica* pods powder on serum lipid profile in hypercholesterolemic rats

The serum lipid profile of hypercholesterolemic rats showed a marked, dose-dependent improvement following supplementation with *A. nilotica* pod ethanolic extract. Triglyceride (TG) levels were significantly higher in the positive control group (186.29 ± 3.72 mg/dL) but decreased notably (p < 0.01) to 156.34, 135.29, and 108.96 mg/dL in the treated groups (G_3_–G_5_), indicating an effective reduction in circulating fat. This decrease may result from the inhibitory effects of *A. nilotica* flavonoids and tannins on hepatic lipid-synthesizing enzymes. Total cholesterol (TC) followed a similar trend, declining from 102.39 ± 2.55 mg/dL in the positive control to 99.65, 96.76, and 94.64 mg/dL in G_3_, G_4_, and G_5_, respectively (p < 0.01). Low-density lipoprotein cholesterol (LDL-C), the major atherogenic fraction, also showed a significant (p < 0.01) dose-dependent decrease from 25.49 ± 0.61 mg/dL in the control group to 24.67, 22.72, and 20.42 mg/dL in the treated groups. Very low-density lipoprotein (VLDL-C) concentrations decreased from 37.26 ± 0.77 mg/dL to 31.27, 27.06, and 21.79 mg/dL in G_3_-G_5_. High-density lipoprotein (HDL-C) levels did not differ significantly among the groups (37.31–39.98 mg/dL) (Table 5).

**TABLE 5:** Effect of different treatments on serum lipid profile in hypercholesterolemic rats.

Serum indices	G_1_	G_2_	G_3_	G_4_	G_5_	F-ratio
HDL (mg/dL)	39.98 ± 1.32^a^	37.31 ± 1.32^a^	37.88 ± 1.32^a^	38.95 ± 1.32^a^	39.25 ± 1.32^a^	1.31^NS^
LDL (mg/dL)	23.25 ± 0.61^bc^	25.49 ± 0.61^a^	24.67 ± 0.61^ab^	22.72 ± 0.61^c^	20.42 ± 0.61^d^	20.83**
TC (mg/dL)	78.09 ± 2.55^c^	102.39 ± 2.55^a^	99.65 ± 2.55^ab^	96.76 ± 2.55^ab^	94.64 ± 2.55^b^	28.01**
TG (mg/dL)	74.32 ± 3.72^e^	186.29 ± 3.72^a^	156.34 ± 3.72^b^	135.29 ± 3.72^c^	108.96 ± 3.72^d^	267.58**
VLDL (mg/dL)	14.86 ± 0.77^e^	37.26 ± 0.77^a^	31.27 ± 0.77^b^	27.06 ± 0.77^c^	21.79 ± 0.77^d^	251.77**

Means having similar alphabets (a-e) do not differ significantly (p-values >0.05), ANOVA, test followed by the *post hoc* Tukey’s HSD, test, HDL (High density lipoproteins), LDL (Low density lipoproteins), VLDL (Very low-density lipoproteins), TC (Total cholesterol), TG (Triglycerides), G_1_; SBD, G_2_; Hypercholesterolemia + SBD, G_3_; Hypercholesterolemia + SBD + ANPE, 250 mg/kg/day, G_4_; Hypercholesterolemia + SBD + ANPE, 500 mg/kg/day, G_5_; Hypercholesterolemia + SBD + ANPE, 750 mg/kg/day; NS, not significant; *p < 0.05; **p < 0.01.

### 3.7 Effect of *A. nilotica* pods on cardiac function biomarkers in hypercholesterolemic rats

The results of *A. nilotica* pod supplementation in hypercholesterolemic rats are presented in Table 6, which shows significant differences in oxidative stress markers and heart functional biomarkers among the experimental groups. Catalase (CAT) and superoxide dismutase (SOD) activity were significantly reduced in the positive control group (G_2_), with values of 8.65 ± 0.50 U/mg protein and 3.87 ± 0.25 U/mg protein, respectively. These activities were significantly and dose-dependently stimulated by *A. nilotica* pod treatment. Group G_4_ (500 mg/kg) showed moderate improvement in both enzymes (CAT: 12.74 ± 0.50 U/mg protein, SOD: 6.96 ± 0.25 U/mg protein), and group G_5_ (750 mg/kg) showed the highest CAT (15.54 ± 0.50 U/mg protein) and SOD (7.79 ± 0.25 U/mg protein). There was also significantly less malondialdehyde (MDA), an oxidative stress biomarker, in the treatment groups, with G5 showing the lowest level at 3.29 ± 0.15 nmol/mL compared to the positive control (6.24 ± 0.15 nmol/mL). The positive control group (G_2_) exhibited the highest levels of cardiac biomarkers (CK: 520.34 ± 9.89 U/L; troponin: 28.72 ± 0.73 ng/mL) and the highest atherogenic and Castelli indices, indicating significant myocardial damage. However, these indicators were reduced in a dose-dependent manner due to *A. nilotica* pod treatment. The greatest decrease in CK (305.56 ± 9.89 U/L), troponin (12.67 ± 0.73 ng/mL), and the atherogenic (1.07 ± 0.03) and Castelli (2.41 ± 0.06) indices were found in Group G_5_, which received the highest dose (750 mg/kg). The other groups G_3_ (250 mg/kg) and G_4_ (500 mg/kg) also had significant decreases (p < 0.01).

**TABLE 6:** Effect of different treatments on oxidative stress markers and cardiac function biomarkers in hypercholesterolemic rats.

Parameters	G_1_	G_2_	G_3_	G_4_	G_5_	F-ratio
CAT (U/mg protein)	18.45 ± 0.50^a^	8.65 ± 0.50^e^	10.45 ± 0.50^d^	12.74 ± 0.50^c^	15.54 ± 0.50^b^	122.71**
SOD (U/mg protein)	8.26 ± 0.25^a^	3.87 ± 0.25^d^	5.81 ± 0.25^c^	6.96 ± 0.25^b^	7.79 ± 0.25^a^	95.91**
MDA (nmol/mL)	2.87 ± 0.15^d^	6.24 ± 0.15^a^	4.02 ± 0.15^b^	3.58 ± 0.15^bc^	3.29 ± 0.15^cd^	158.31**
CK (U/L)	257.67 ± 9.89^e^	520.34 ± 9.89^a^	439.65 ± 9.89^b^	377.82 ± 9.89^c^	305.56 ± 9.89^d^	223.36**
Troponin (ng/mL)	8.19 ± 0.73^e^	28.72 ± 0.73^a^	21.51 ± 0.73^b^	17.63 ± 0.73^c^	12.67 ± 0.73^d^	237.23**
Atherogenic Index (ATH)	0.95 ± 0.03^e^	1.68 ± 0.03^a^	1.48 ± 0.03^b^	1.28 ± 0.03^c^	1.07 ± 0.03^d^	177.25**
Castelli Index	1.95 ± 0.06^d^	2.74 ± 0.06^a^	2.63 ± 0.06^ab^	2.48 ± 0.06^bc^	2.41 ± 0.06^c^	44.11**

Means having similar alphabets (a-e) do not differ significantly (p-values >0.05), ANOVA, test followed by the *post hoc* Tukey’s HSD, test, Catalase (CAT), Superoxide dimutase (SOD), malondialdehyde (MDA), G_1_; SBD, G_2_; Hypercholesterolemia + SBD, G_3_; Hypercholesterolemia + SBD + ANPE, 250 mg/kg/day, G_4_; Hypercholesterolemia + SBD + ANPE, 500 mg/kg/day, G_5_; Hypercholesterolemia + SBD + ANPE, 750 mg/kg/day; significant: **p < 0.01.

### 3.8 Effect of *A. nilotica* pods powder on kidney and liver function tests and protein profile on hypercholesterolemic rats

The results in Table 7 show that liver function and protein profile of hypercholesterolemic rats supplemented with *A. nilotica* pod powder were significantly improved. The levels of serum urea (47.24 ± 1.05 mg/dL vs. 27.10 ± 1.05 mg//dL), creatinine (1.22 ± 0.03 mg/dL vs. 0.78 ± 0.03 mg/dL), and uric acid (2.28 ± 0.07 mg/dL vs. 1.65 ± 0.07 mg/dL) were significantly different in the hypercholesterolemic control group (G_2_) and normal control group (G_1_) with p-values <0.01. All these indicators were significantly lowered in a dose-dependent manner after administration of *A. nilotica* pod extract. Particularly, group G_5_ (750 mg/kg) had the lowest serum urea level (31.25 ± 1.05 mg/dL), which was significantly lower than that of the positive control group (47.24 ± 1.05 mg/dL) (p < 0.01). Similarly, serum uric acid concentration decreased in a dose-dependent manner. The creatinine levels of the treated groups also decreased dramatically, with group G5 having the lowest level (0.83 ± 0.03 mg/dL), which was significantly lower than that of the positive control group (1.22 ± 0.03 mg/dL) (p < 0.01). For liver function, compared to the normal control group (G1), the hypercholesterolemic control group (G_2_) had markedly higher levels of ALP (224.30 U/L), ALT (48.25 U/L), and AST (135.25 U/L) (p < 0.01). *A. nilotica* pod powder supplementation reduced the levels of these liver enzymes (ALP, ALT, and AST) in a dose-dependent manner, with ALP having a maximum decrease. There were also significant improvements in the albumin and bilirubin levels in the treated groups, with G_5_ having the highest albumin levels (4.12 ± 0.10 g/dL, p < 0.01) and lowest bilirubin levels (0.65 ± 0.02 mg/dL). The findings suggest that *A. nilotica* has a hepatoprotective effect, which improves the protein profiles and liver function in cholesterol-increased rats.

**TABLE 7:** Effect of different treatments on liver function and protein profile parameters in hypercholesterolemic rats.

Parameters	G_1_	G_2_	G_3_	G_4_	G_5_	F-ratio
Urea (mg/dL)	27.10 ± 1.05^e^	47.24 ± 1.05^a^	41.86 ± 1.05^b^	37.24 ± 1.05^c^	31.25 ± 1.05^d^	116.82**
Uric Acid (mg/dL)	1.65 ± 0.07^b^	2.28 ± 0.07^a^	2.19 ± 0.07^a^	2.09 ± 0.07^a^	1.84 ± 0.07^b^	29.51**
Creatinine (mg/dL)	0.78 ± 0.03^d^	1.22 ± 0.03^a^	1.02 ± 0.03^b^	0.92 ± 0.03^c^	0.83 ± 0.03	91.10**
ALP (U/L)	141.20 ± 5.57^d^	224.30 ± 5.57^a^	196.10 ± 5.57^b^	179.50 ± 5.57^bc^	165.30 ± 5.57^c^	63.49**
ALT (U/L)	29.95 ± 0.84^d^	48.25 ± 0.84^a^	43.35 ± 0.84^b^	36.56 ± 0.84^c^	31.34 ± 0.84^d^	173.59**
AST (U/L)	75.10 ± 1.95^e^	135.25 ± 1.95^a^	117.20 ± 1.95^b^	102.30 ± 1.95^c^	92.30 ± 1.95^d^	278.28**
Albumin (g/dL)	4.05 ± 0.10^a^	3.50 ± 0.10^c^	3.68 ± 0.10^bc^	3.98 ± 0.10^ab^	4.12 ± 0.10^a^	13.35**
Bilirubin (mg/dL)	0.55 ± 0.02^d^	0.89 ± 0.02^a^	0.84 ± 0.02^a^	0.72 ± 0.02^b^	0.65 ± 0.02^c^	74.15**
Globulin (g/dL)	3.42 ± 0.12^a^	3.31 ± 0.12^a^	3.21 ± 0.12^a^	3.31 ± 0.12^a^	3.38 ± 0.12^a^	1.00^NS^
Total Protein (g/dL)	7.60 ± 0.32^a^	7.19 ± 0.32^b^	7.22 ± 0.32^b^	7.18 ± 0.32^b^	7.14 ± 0.32^b^	5.29**

Means having similar alphabets (a-e) do not differ significantly (p-values >0.05), ANOVA, test followed by the *post hoc* Tukey’s HSD, test, Alanine transaminase (ALT), Aspartate transaminase (AST), Alkaline phosphatase (ALP), G_1_; SBD, G_2_; Hypercholesterolemia + SBD, G_3_; Hypercholesterolemia + SBD + ANPE, 250 mg/kg/day, G_4_; Hypercholesterolemia + SBD + ANPE, 500 mg/kg/day, G_5_; Hypercholesterolemia + SBD + ANPE, 750 mg/kg/day; NS, not significant; **p < 0.01.

### 3.9 Effect of *A. nilotica* pods powder on differential counts in hypercholesterolemic rats

Supplementary Table S3 of the differential leukocyte analysis showed that the hypercholesterolemic control group (1.13%) had a significantly higher number of basophils than the normal control group (0.20%), indicating increased inflammation in the hypercholesterolemic rats (p < 0.01). The hypercholesterolemic population exhibited a greater eosinophil count (2.36%) than the normal population (1.50%). However, eosinophil counts reduced proportionally to the dose of *A. nilotica* treatment. The group receiving the highest dose (G_5_) showed the lowest eosinophil counts, indicating attenuation of inflammation with *A. nilotica* supplementation. The percentage of lymphocytes in all groups was relatively stable, indicating that *A. nilotica* supplementation had a minimal effect on adaptive immunity. Neutrophil percentages were slightly but significantly lower (p < 0.05) in the treated groups (31.33%–32.47%) compared with the normal control group (36.35%) and the hypercholesterolemic control group (32.25%). Based on these results, *A. nilotica* can be an anti-inflammatory agent in individuals with high cholesterol levels.

### 3.10 Histopathological findings in liver tissues

**FIGURE 2:**
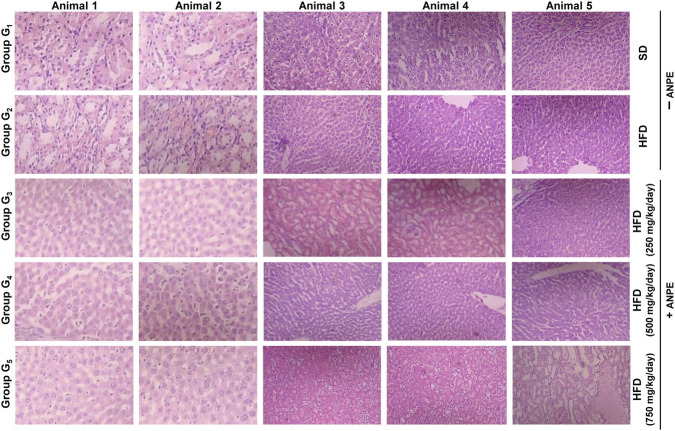
Liver histopathological architecture in control, disease, and treated groups. Representative hematoxylin and eosin (H&E)-stained liver sections from rats fed a standard basal diet (G_1_), high-fat diet control (G_2_), or high-fat diet plus ANPE at 250, 500, or 750 mg/kg body weight per day (G_3_–G_5_). G_2_ shows marked hepatic injury with hepatocyte disorganization, sinusoidal dilation, central vein congestion, inflammatory cell infiltration, and steatosis compared with the normal architecture in G_1_. ANPE treatment attenuates these lesions in a dose-dependent manner, with partial improvement in G_3_-G_4_ and near-normal hepatic architecture in G_5_, consistent with the semi-quantitative histopathology scores in Supplementary Table S4.

Histopathological analysis of liver tissues revealed clear differences between the normal control, hypercholesterolemic control, and treated groups. In the normal control group (G_1_), hepatocytes, liver architecture, and central veins appeared normal, with no signs of inflammation, sinusoidal dilatation, or fat accumulation (grade 0 for all parameters) (Supplementary Table S4). In the hypercholesterolemic control group (G_2_), there was slight disorganization of liver tissue and inflammation, with a grade 1 score observed across all parameters, indicating the onset of liver stress and damage due to fat overload. In group G_3,_ there was evident liver damage, inflammation, and dilation, with each parameter graded as 2. These changes indicate steatohepatitis, a consequence of a high-fat diet (Figure 2). In G_4_, partial recovery was observed. The organization of hepatocytes and vascular structures showed improvement, mostly reverting to grade 1. However, fat accumulation increased to grade 2, and there were no changes in inflammation, with lipid clearance being slower due to metabolic disturbances. On the other hand, G5 (Treated 4) exhibited complete restoration of liver structure. All histopathological parameters returned to normal (grade 0), indicating a hepatoprotective effect. The liver tissue was comparable to that of the control group. The Kruskal-Walli’s test was used to statistically assess significant differences between groups (p < 0.05).

### 3.11 Histopathological evaluation of kidney tissues

**FIGURE 3:**
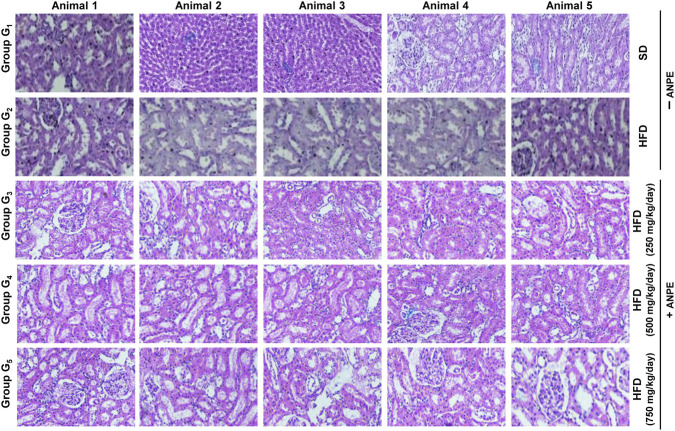
Kidney histopathological architecture in control, disease, and treated groups. Representative H&E-stained kidney sections from rats fed a standard basal diet (G_1_), high-fat diet control (G_2_), or high-fat diet plus ANPE at 250, 500, or 750 mg/kg body weight/day (G_3_–G_5_). G_1_ exhibits normal glomeruli and renal tubules, with no evidence of inflammation or cast formation, whereas G_2_ shows pronounced glomerular damage, tubular degeneration, interstitial inflammation, and intraluminal casts. ANPE supplementation reduces these pathological changes in a dose-dependent fashion: G_3_ and G_4_ show moderate improvement with reduced degeneration and inflammation, while G_5_ displays largely preserved renal architecture with minimal inflammatory infiltrates and absence of casts, in agreement with the quantitative scoring in Supplementary Table S5.

Microscopic examination of kidney tissue revealed distinct structural and health differences among the experimental groups. In the control group (G_1_), the kidney anatomy appeared entirely normal (Supplementary Table S5). The glomeruli were undamaged, the tubules were in good condition, and there were no signs of inflammation or casts, indicating healthy kidney function (Figure 3). The G_2_ group showed moderate structural damage. Histopathological analysis indicated Grade 2 injury in the glomeruli, noticeable degeneration of the tubular structure, interstitial inflammation, and slight cast formation. These are common characteristics of nephropathy, where chronic metabolic stress and oxidative damage gradually impair nephron structure and lead to kidney dysfunction. The G_3_ group, which received treatment with *A. nilotica* pod extract, showed promising results. Histopathological changes were minimal, and notably, there was no sign of cast formation. In G_4_ (Treated Group 2), there was a degree of recovery, though it was not particularly pronounced. While glomerular damage and inflammation were less severe compared to G_2_, there was still moderate tubular atrophy and some remaining cast formation. This partial recovery might be attributed to either an insufficient dosage or an inadequate duration of treatment to fully reverse the damage. The most favorable outcomes were observed in G_5_ (Treated Group 3), where the highest dose of ANPE was administered. In this group, the kidney tissue appeared largely normal, with intact glomeruli and tubules, minimal inflammatory infiltration, and no casts. This suggests a significant nephroprotective effect at high extract concentrations, likely due to the abundance of antioxidants and anti-inflammatory phytochemicals in the plant.

### 3.12 In silico identification of putative modulators of oxidosqualene cyclase

The docking simulations evaluated the binding affinities of ten *A. nilotica* pod compounds against oxidosqualene cyclase (OSC) (Supplementary Table S6). Larger negative docking scores, ranging from −5.9 to −9.7, indicate a stronger anticipated binding. The most favorable binder was β-sitosterol with a docking score of −9.7, showing a strong affinity for the OSC active site. In contrast, simple phenolics like gallic acid (score of −5.9) had the lowest scores, suggesting weaker interactions. Sterol and triterpene structures (β-sitosterol, β-amyrin, and betulin) performed better overall than polyphenols and small phenols, suggesting that the larger hydrophobic cores of sterols allow for greater binding in the OSC pocket.

**FIGURE 4:**
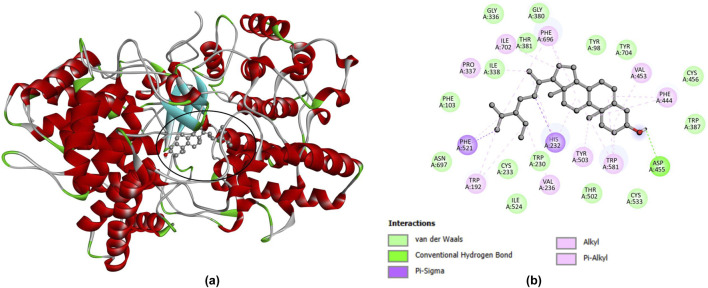
Molecular docking study for the most efficient compound β-Sitosterol **(a)** β-Sitosterol docked OSC **(b)** Interaction profile. Molecular docking of β-sitosterol with human oxidosqualene cyclase (OSC, PDB ID access. no.: 1W6J). **(a)** Three-dimensional view of β-sitosterol (ball-and-stick representation) bound deep within the active-site cavity of OSC (cartoon representation), illustrating its insertion into the hydrophobic pocket and orientation of the 3-hydroxyl group toward a polar region of the binding site. **(b)** Two-dimensional ligand-receptor interaction diagram showing hydrogen bonds, van der Waals contacts, and hydrophobic (alkyl/π-alkyl and π-sigma) interactions formed between β-sitosterol and key OSC residues, consistent with the favorable docking score (−9.7) reported in Supplementary Table S6.

The docking pose of β-sitosterol-OSC is depicted in Figure 4. The β-sitosterol molecule occupies a deep position within the binding cavity, with nonpolar OSC side chains surrounding its hydrophobic sterol rings. The polar 3-hydroxyl group of β-sitosterol points towards a polar region of the pocket, potentially forming a hydrogen bond with a polar amino acid on OSC. The extended aliphatic tail of β-sitosterol fits into an adjacent hydrophobic groove, establishing van der Waals contacts with residues like phenylalanine or leucine. The docking model suggests the presence of at least one stabilizing hydrogen bond and several hydrophobic contacts in the interaction profile (Figure 4b). All of these components work together to secure β-sitosterol in a preferred conformation within the active region of the enzyme.

### 3.13 Molecular dynamics study

**FIGURE 5:**
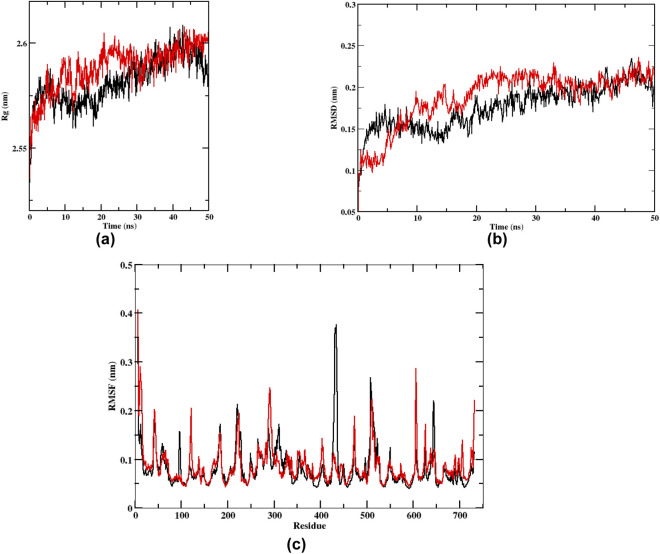
Molecular dynamics (MD) analysis of human OSC in its apo form (black traces) and in complex with β-sitosterol (red traces) over 50 ns of simulation in explicit solvent. **(a)** Time evolution of the radius of gyration (Rg) showing comparable and stable global compactness of the apo and ligand-bound protein. **(b)** Backbone root-mean-square deviation (RMSD) relative to the initial structure, indicating rapid equilibration and low fluctuations, consistent with a stable OSC/β-sitosterol complex. **(c)** Per-residue root-mean-square fluctuation (RMSF) profile demonstrating limited flexibility of most residues and reduced motion of active-site residues lining the β-sitosterol binding pocket, supporting a structurally robust complex.

The stability and conformational dynamics of the β-sitosterol-OSC complex are revealed by molecular dynamics (MD) simulations conducted over a period of 50 ns. The RMSD plot (Figure 5a) shows that the protein-ligand complex equilibrates quickly: after a brief initial rise (approximately first 5–10 ns), the backbone RMSD levels off and fluctuates narrowly for the remainder of the run. With only slight variations (about 0.2–0.3 nm from the initial structure), the plateau shows that β-sitosterol is still firmly attached and that the protein structure is stable overall. This low, stable RMSD suggests the docked pose corresponds to a true energy minimum rather than an artifact. Figure 5b shows residue-wise flexibility in the RMSF profile. Low variations (RMSF ∼0.1–0.2 nm) are seen in most residues, suggesting a largely stiff complex. Importantly, residues lining the β-sitosterol binding pocket show minimal movement, confirming that the active site remains well-structured throughout the simulation. In other words, the binding of β-sitosterol does not cause the core of the enzyme to become too flexible. The radius of gyration (Rg) (Figure 5c) remains essentially constant over time (with only minor fluctuations), reflecting a stable overall compactness of the protein. The absence of any structural expansion or collapse suggests that the protein does not experience significant unfolding or structural rearrangement during the intricate simulation. Together, the RMSD, RMSF, and Rg analyses indicate that the β-sitosterol–OSC complex is structurally robust: after initial adjustment, the ligand stays in place and the enzyme maintains its global fold.

## 4 Discussion

The pods of *A. nilotica* contain antioxidants that are important for promoting health and healing. Antioxidants include tannins, flavonoids, and phenolic compounds, which have strong anti-inflammatory, antioxidative, and curative effects and are used to treat various medical conditions (Saeedi et al., 2020). Over the past few years, the incidence of hyperlipidemia has increased worldwide, and the major factor behind this tendency is an improper diet, which means consuming a considerable portion of processed food and saturated fat (Zahra et al., 2020). Plant-based remedies have become attractive solutions owing to the new trend of seeking natural replacements to enhance lipids and curb oxidative stress (Sene et al., 2023). One of these is the *A. nilotica* pods with high antioxidant potential, which has been shown to be useful in enhancing general health, especially in the management of cardiovascular health (Jain et al., 2025). In the modern age, the need to enhance cardiac function and lower lipid levels is necessary to sustain high levels of heart function because of the continuously rising cholesterol levels worldwide (Khalaf et al., 2023).

*A. nilotica* pods have been found to possess great potential for decreasing total cholesterol, decreasing LDL and augmenting the atherogenic index, thereby, rendering a potent plant in augmenting cardiovascular activity (Kumari and Swer, 2025). It is also necessary to state that *A. nilotica* supplementation has been observed to raise the levels of HDL cholesterol, which is critical in reducing the occurrence of cardiovascular diseases (Panda et al., 2025). Additionally, the pods are dose-dependent, with effects of increasing lipid metabolism and inhibiting oxidative stress as an alternative to pharmaceutical methods in managing hyperlipidemia and other health complications (Ahmed et al., 2024). The ability of the plant to alter lipid levels, particularly by increasing HDL and decreasing LDL, makes it a possible candidate for the treatment of cardiovascular and metabolic diseases (Saeedi et al., 2020). The TPC values indicate that *A. nilotica* pods are rich in phenolic compounds, especially in the ethanol extracts. Ethanol was the best solvent to employ in the process of extracting phenolic chemicals in *A. nilotica* pods since the TPC was the greatest in the ethanol extract. These phenols are known to be anti-inflammatory, anticancer, and antioxidant, which validates the health potential of the plant. The FRAP assay results also provide additional evidence that the reducing power of ethanol is the greatest, and thus it is the best choice of solvent for extracting antioxidants. The ethanol extract had the greatest reducing power in this test, which determines the capacity of a substance to transform ferric ions into ferrous ions. A high reducing power of the water extract was also observed, implying that the pods contained hydrophilic antioxidant chemicals. The comparatively lower reducing power of the methanol and acetone extracts may be attributed to differences in the solubility and ferric-reducing capacity of their bioactive compounds. This agrees with the results of others, who reported that ethanol and aqueous extracts of *A. nilotica* were applicable in the determination of strong antioxidant activity (Agravat et al., 2025; Riasat et al., 2024). However, our study lack of direct batch-specific LC-MS profiling and standardization.

As demonstrated in the current research, the ethanol extract of *A. nilotica* pods showed the highest radical scavenging activity in the DPPH assay, which evaluates how well a sample can neutralize free radicals. This suggests that *A. nilotica* pod extracts prepared with ethanol are rich in antioxidants. The water extract also exhibited a strong scavenging effect, indicating that *A. nilotica* contains water-soluble antioxidants that can effectively eliminate free radicals. Despite a lower radical scavenging capacity than the ethanolic extract, the methanolic and acetone extracts also showed antioxidant potential with different solubility characteristics. This highlights the importance of selecting the appropriate solvent for extracting specific bioactive compounds (Sene et al., 2023; Kumar et al., 2022). Both ethanol and methanol were found to be effective solvents for extracting antioxidants from *A. nilotica* pods, making them a promising source for pharmaceutical and nutraceutical products to combat oxidative stress.

The study revealed that supplementation with *A. nilotica* powder significantly and dose-dependently improved the serum lipid profile of hypercholesterolemic rats. Notably, a reduction in triglycerides (TG) levels from 186.29 mg/dL in the control group to 108.96 mg/dL in the G_5_ group indicated a lipid-lowering effect. *A. nilotica* also decreased LDL-C, VLDL-C, and total cholesterol (TC) levels in treated rats, suggesting a role in lipid metabolism regulation, possibly by inhibiting lipid-producing enzymes. These findings align with previous studies (Omara et al., 2012; Abuelgassim, 2013; Zakernezhad et al., 2021), which reported similar reductions in serum lipids with *A. nilotica* administration in animal models. HDL-C levels remained stable, indicating that *A. nilotica* does not decrease healthy lipoprotein concentrations. This suggests that *A. nilotica* could be used as a therapeutic agent to modify lipid profiles without negatively affecting HDL-C levels.

This study also demonstrated the effects of *A. nilotica* on various hematological parameters. The erythrocyte sedimentation rate (ESR), an important indicator of inflammation, was drastically reduced in a dose-dependent manner from 2.76 mm/h to 1.58 mm/h, especially in the highest dose group (G_5_). This reduction indicates a decrease in inflammation associated with lipid disproportionation. Similarly, the Hematocrit (HCT) values of the treated rats partially returned to normal, with G_5_ showing 41.25, indicating its ability to protect hydration and red cell volume. The level of hemoglobin (Hb) was not affected, showing that *A. nilotica* did not negatively impact erythropoiesis. This increase in hemoglobin content per red blood cell (reflected by higher mean corpuscular hemoglobin, MCH) enhances the oxygen-carrying capacity of the blood, which is beneficial in dyslipidemic conditions. These findings align with previous studies, demonstrating that plant-derived antioxidants help restore hematological balance under dyslipidemic conditions (Devaki et al., 2012; Olorunnisola et al., 2016). As shown in the study results, *A. nilotica* significantly reduced the number of basophils and eosinophils, indicating reduced inflammation. The basophil counts of the positive control group were much higher (1.13%), but were reduced by *A. nilotica* therapy to almost normal ranges (0.67% in G_3_-G_5_, 0.62%, and 0.63%). This reduction suggests that *A. nilotica* lowers inflammation potentially by inhibiting mast cell degranulation and histamine release as shown in a model of allergic dermatitis (Ibrahim et al., 2025). Other indicators of the anti-inflammatory and antioxidant properties of *A. nilotica* include the reduction of eosinophils, indicating the attenuation of lipid peroxidation-induced inflammation, consistent with earlier reports (Iftikhar et al., 2022). Neutrophil counts also reflected reduced oxidative stress, similar to trends reported with garlic extract (Anyanwu et al., 2023).

The study data showed that *A. nilotica* had a significant and positive effect on the livers of hypercholesterolemic rats. The positive control group showed significantly increased serum liver enzymes, including ALP, ALT, and AST, signifying hepatic damage. However, the enzymes were reduced with the dose, with a significant reduction at the highest dose of *A. nilotica* supplement (750 mg/kg BW). Further indication of hepatoprotective properties is demonstrated by the increase in albumin levels and reduction in bilirubin levels. The atherogenic index (ATH) and Castelli index findings indicate improved lipid handling and reduced cardiovascular risk, supporting previous studies that reported similar effects of *A. nilotica* on hepatic and lipid metabolism in obesity-related animal models (Khalaf et al., 2023). As shown in the study, supplementation with *A. nilotica* produced a significant dose-dependent reduction in BW, with the highest dose group (G_5_) at 181.29 g. Similarly, it was found that this reduction is in tandem with the anti-obesity effects of *A. nilotica*, which correlates with enhanced lipid oxidation and AMPK regulation (Khalaf et al., 2023). In addition, the liver weights of the treated rats decreased, indicating that it was protective against hepatic lipid infiltration, as reported in a previous study using *Tamarindus indica* (Kuddus et al., 2020). Moreover, the weight of the treated groups returned to normal in the spleen, which was a sign of reduced inflammation (Iftikhar et al., 2022).

Moreover, this study demonstrated that antioxidant enzyme activities were significantly enhanced with *A. nilotica* supplementation. High levels of oxidative stress were observed in the hypercholesterolemic group, as evidenced by the decreased CAT and SOD values compared to normal levels. Lipid peroxidation, indicated by altered MDA levels, was also reduced with *A. nilotica* treatment, particularly at higher dosages (750 mg/kg). These results suggest that *A. nilotica* may have potent antioxidant properties, likely due to its rich content of flavonoids, tannins, and polyphenols that act as free radical scavengers, protecting cellular membranes from oxidative damage. This is consistent with findings of others (Khalaf et al., 2023), who reported decreased MDA levels and restored antioxidant enzyme activity in high-fat diet models treated with *A. nilotica* stem bark extract. Additionally, the antioxidant properties of *A. nilotica* green pod extracts have been shown to be robust (Kuddus et al., 2020). Overall, these findings highlight that *A. nilotica* exerts strong antioxidant and hepatoprotective effects against oxidative damage induced by cholesterol.

The study found that members of the hypercholesterolemic control group had significant higher levels of troponin and creatine kinase (CK) in their serum, which may indicate heart damage. The improvement in cardiac biomarkers was attributed to the potent antioxidant and anti-lipidemic effects of *A. nilotica*. Its flavonoids and tannins likely reduce cardiac oxidative stress, stabilize the myocardial membrane, and inhibit lipid peroxidation in cardiac tissue. These findings align with a previous report (Khalaf et al., 2023), which found a positive effect of *A. nilotica* stem bark extract on cardiac and hepatic function. It also confirms previous findings (Ram et al., 2014) that observed a reduction in lipid peroxidation and improvement in antioxidant activity in hypercholesterolemic animals. Overall, *A. nilotica* has great cardioprotective potential due to the reduction of oxidative and lipid-induced myocardial injury.

This study showed a significant improvement in renal function after administration of *A. nilotica*. The levels of urea, creatinine, and uric acid in the hypercholesterolemic control group increased, indicating renal impairment. However, these indicators were drastically reduced with *A. nilotica* treatment, particularly at the highest dose (750 mg/kg), showing a dose-dependent effect. The renoprotective properties of *A. nilotica* can be attributed to its high concentration of phytochemicals, such as flavonoids, tannins, and polyphenols, which have free radical scavenging activity, prevent inflammatory mediators, and stabilize renal cell membranes. These results align with previous research showing improved renal and hepatic profiles in hyperlipidemic rats treated with *A. nilotica* extracts (Khalaf et al., 2023), as well as a reduction in gentamicin-induced nephrotoxicity through antioxidant and anti-inflammatory effects (Goel et al., 2025). These findings suggest that *A. nilotica* is an effective nephroprotectant against hypercholesterolemia-induced renal dysfunction by restoring oxidative balance and improving metabolic homeostasis. *A. nilotica* also significantly reduces the Castelli index, a measure of a lipoprotein’s atherosclerosis-causing potential, by lowering LDL and total cholesterol levels and stabilizing HDL. This beneficial effect is attributed to the presence of polyphenols, flavonoids, and tannins in *A. nilotica*, which prevent LDL oxidation and enhance lipid metabolism. Similar hypolipidemic and cardioprotective effects have been observed with *A. nilotica* leaf extract in diabetic rats (Asad et al., 2015).

The combined molecular docking and dynamics results present a coherent picture: β-sitosterol, among the compounds computed from *A. nilotica*, has the highest predicted affinity for OSC and forms a stable complex under dynamic simulation. β-sitosterol was found to be the top binder in the docking investigation, suggesting it can efficiently occupy the OSC active site. The subsequent MD simulations confirmed the continued tight binding of the complex, showing little structural drift. β-sitosterol and related ligands may decrease the synthesis of lanosterol and downstream cholesterol by binding OSC. The experimental finding that *A. nilotica* extract reduces serum cholesterol and LDL in hypercholesterolemic animals is consistent with this molecular mechanism. Reduced cholesterol synthesis would likely lead to decreased sterol-driven inflammatory signaling. Cholesterol and its metabolites can modulate inflammatory pathways; thus, OSC inhibition could contribute to the anti-inflammatory effects reported for β-sitosterol and related compounds (Darden et al., 1993). These findings demonstrate the importance of *A. nilotica* phytochemicals from a therapeutic perspective. One lead compound that shows special promise is β-sitosterol (Asad et al., 2015; Kumari et al., 2014). Therefore, β-sitosterol’s high affinity to OSC, coupled with its known physiological effects, reinforces the case for its role in managing hypercholesterolemia and inflammation through a molecular mechanism. The current study relies on systemic lipid markers and histopathological outcomes to assess efficacy. In the absence of direct lanosterol pathway assays, the *in silico* findings provide a basis for future biochemical validation. Recent investigations have also reported that OSC enzymes are involved in oxidative stress and disease pathophysiology through cholesterol biosynthesis pathways (Allam et al., 2025), which supports the biological relevance of the present *in silico* findings. Previous studies have also highlighted the cholesterol biosynthesis pathway as a promising therapeutic target, particularly in relation to angiogenesis, cancer progression, cholesterol metabolism, and metabolic regulation (Maione et al., 2015; Slight and Hyder, 2022).

These findings are supported by earlier studies that observed improvements in liver histology, noting reduced steatosis and inflammation in rats treated with *A*. *nilotica* pod extract (Idrees et al., 2024). Similarly, other studies have highlighted the hepatoprotective effects of *Acacia catechu*, where liver structure was restored in models challenged with a high-fat diet and toxins or acetaminophen (Lakshmi et al., 2018). In group G_5_, the complete restoration of normal liver cell arrangement, absence of inflammation, and elimination of fat deposits indicate the significant regenerative potential of Acacia pod extracts when administered in high doses. This effect is likely due to the inhibition of lipid peroxidation and the down-regulation of inflammatory mediators, as shown in concurrent biochemical studies.

These results are consistent with recent research findings that have demonstrated the significant impact of *A*. *nilotica* pod extract in reducing oxidative stress and improving kidney histology in diabetic rats. The protective effects can be potentially attributed to bioactive molecules such as tannins, flavonoids, and polyphenols (Omara et al., 2012). These compounds likely play a similar role in tissue repair, as evidenced by the observations in groups G_3_ and G_5_. *Acacia* pod extracts have been shown to prevent glomerular hypertrophy, tubular necrosis, and inflammatory infiltration in kidney toxicosis models (Omara et al., 2012), which aligns with the structural improvements observed in this study, including the absence of casts in group G_5_. Previous studies have also highlighted the nephroprotective properties of *A. nilotica* in a gentamicin-induced nephrotoxicity model (Goel et al., 2025). In this model, *A. nilotica* extracts suppressed caspase signaling pathways, increased antioxidant mechanisms, and inhibited oxidative stress, inflammation, and apoptosis in a dose-dependent manner (Goel et al., 2025).

## 5 Conclusion

In conclusion, *A. nilotica* pod extracts show promising therapeutic effects in diet-induced hypercholesterolemia and its associated complications in rats. Among the extracts examined, the ethanolic extract (ANPE) exhibited the strongest hypocholesterolemic properties, with a significant decrease in total cholesterol and LDL levels and a trend toward higher HDL levels. The ethanolic extract was effective in dose-dependently reducing serum triglycerides, VLDL, and renal markers such as urea and creatinine. The ethanolic extract also showed moderate improvement in inflammatory and oxidative stress markers, supporting its anti-inflammatory potential. The ethanolic extract led to improved hematological values, especially an increase in red blood cell counts and hematocrit. Histopathological analyses proved the protective effect of the extracts on liver and kidney tissues. These findings demonstrate the antihypercholesterolemic, hepatoprotective, nephroprotective, and hematinic properties of *A. nilotica* pods, implying their usefulness as natural therapeutic agents in the management of cardiovascular and metabolic diseases. Future studies should directly compare ANPE with standard hypolipidemic drugs to benchmark its efficacy and safety against established therapies. Nevertheless, additional research, including clinical trials, is advised to confirm these findings and investigate their use in human health. Computational analyses identify β-sitosterol, a major pod constituent, as a potential OSC inhibitor, offering a theoretical framework that warrants future experimental validation using specific pathway markers. The findings underscore the potential of *A. nilotica* pod compounds as putative natural OSC inhibitors, which may contribute to their observed lipid-lowering and anti-inflammatory benefits. However, these conclusions are based on a relatively small sample size and a single animal model, and extrapolation to humans should be made with caution. Moreover, the study did not include a standard hypolipidemic drug control, which limits direct comparison with existing therapies. Finally, we have not standardized our *A. nilotica* extracts in terms of batch-specific LC-MS profiling, which however, is a common issue when utilizing plant extracts in biomedical research.

## Data Availability

The original contributions presented in the study are included in the article/Supplementary Material, further inquiries can be directed to the corresponding authors.
