# Supplementary material for: Investigating the phytotherapeutic efficacy of Acacia nilotica pod ethanolic extract in modulating lipid profile, oxidative stress and inflammation in diet-induced hypercholesterolemia: in vivo and in silico insights
Source: Front Pharmacol. 2026 May 7;17:1831586. doi: 10.3389/fphar.2026.1831586 (PMC13190190; doi:10.3389/fphar.2026.1831586)
Supplement: Supplementary file 1 [file Supplementaryfile1.docx]

Supplementary Material

**Supplementary Table 1: Proximate composition of *A. nilotica* pods**

| **Parameters** | **Compositions g/100 g** |
| --- | --- |
| Moisture | 9.50±0.48 |
| Fat | 0.12±0.01 |
| Protein | 6.00±0.30 |
| Crude fiber | 18.40±0.15 |
| Ash | 6.70±0.34 |
| Nitrogen-free extract (NFE) | 41.57±0.40 |

**Supplementary Table 2: Effect of different treatments on hematological parameters in hypercholesterolemic rats**

| **Parameter** | **G_1_** | **G_2_** | **G_3_** | **G_4_** | **G_5_** | **F-Ratio** |
| --- | --- | --- | --- | --- | --- | --- |
| ESR (mm/hr) | 0.75±0.06^e^ | 2.76±0.06^a^ | 2.36±0.06ᵇ | 1.92±0.06ᶜ | 1.58±0.06ᵈ | 286.77** |
| HCT (%) | 44.52±1.20^a^ | 40.12±1.20^b^ | 40.50±1.20ᵇ | 40.95±1.20ᵇ | 41.25±1.20ᵃᵇ | 4.31* |
| Hb (g/dL) | 11.10±0.41ᵃ | 9.95±0.41ᵃ | 10.34±0.41ᵃ | 10.65±0.41ᵃ | 10.89±0.41ᵃ | 2.40^NS^ |
| MCH (pg) | 14.78±0.37ᵇ | 14.80±0.37ᵇ | 16.03±0.37ᵃ | 15.50±0.37ᵃᵇ | 15.25±0.37ᵃᵇ | 4.04* |
| MCHC (g/dL) | 24.93±0.65ᵃ | 24.80±0.65ᵃ | 25.53±0.65ᵃ | 26.01±0.65ᵃ | 26.40±0.65ᵃ | 2.22^NS^ |
| MCV (fL) | 59.28±1.29ᵃᵇ | 59.70±1.29ᵃᵇ | 62.79±1.29ᵃ | 59.61±1.29ᵃᵇ | 57.77±1.29ᵇ | 3.99* |
| RBC (×10⁶/µL) | 7.51±0.18^a^ | 6.72±0.18^bc^ | 6.45±0.18ᶜ | 6.87±0.18ᵇᶜ | 7.14±0.18ᵃᵇ | 9.90** |
| WBC (×10³/µL) | 8.32±0.27^c^ | 9.55±0.27^a^ | 9.25±0.27ᵃᵇ | 8.95±0.27ᵃᵇᶜ | 8.45±0.27ᵇᶜ | 7.46** |

Means having similar alphabets do not differ significantly (p-values > 0.05), ANOVA test followed by the post-hoc Tukey’s HSD test, RBC: Red blood cell, Hb: Hemoglobin, HCT: Hematocrit,, MCV: Mean corpuscular volume, MCH: Mean corpuscular hemoglobin, MCHC: Mean corpuscular hemoglobin concentration, G_1_; SBD, G_2_; Hypercholesterolemia + SBD, G_3_; Hypercholesterolemia + SBD + ANPE 250 mg/kg/day, G_4_; Hypercholesterolemia+ SBD+ ANPE 500 mg/kg/day, G_5_; Hypercholesterolemia+ SBD+ ANPE 75 0mg/kg/day; NS, not significant; *, p<0.05; **, p<0.01.

**Supplementary Table 3: Effect of different treatments on differential count in hypercholesterolemic rats**

| **Hematological Indices** | **G_1_** | **G_2_** | **G_3_** | **G_4_** | **G_5_** | **F-Ratio** |
| --- | --- | --- | --- | --- | --- | --- |
| Basophil (%) | 0.20±0.02^c^ | 1.13±0.02^a^ | 0.67±0.02ᵇ | 0.62±0.02ᵇ | 0.63±0.02ᵇ | 571.96** |
| Eosinophil (%) | 1.50±0.05^c^ | 2.36±0.05^a^ | 2.09±0.05ᵇ | 2.06±0.05ᵇ | 1.98±0.05ᵇ | 87.07** |
| Lymphocyte (%) | 59.25±1.47ᵃ | 61.14±1.47ᵃ | 61.45±1.47ᵃ | 61.90±1.47ᵃ | 62.45±1.47ᵃ | 1.36^NS^ |
| Monocytes (%) | 2.70±0.08^d^ | 3.12±0.08^c^ | 3.32±0.08ᵇᶜ | 3.47±0.08ᵃᵇ | 3.61±0.08ᵃ | 37.96** |
| Neutrophil (%) | 36.35±0.96^a^ | 32.25±0.96^b^ | 32.47±0.96ᵇ | 31.95±0.96ᵇ | 31.33±0.96ᵇ | 8.69** |

Means having similar alphabets do not differ significantly (p-values > 0.05), ANOVA test followed by the post-hoc Tukey’s HSD test, RBC: Red blood cell, Hb: Hemoglobin, HCT: Hematocrit, MCV: Mean corpuscular volume, MCH: Mean corpuscular hemoglobin, MCHC: Mean corpuscular hemoglobin concentration, G_1_; SBD, G_2_; Hypercholesterolemia + SBD, G_3_; Hypercholesterolemia + SBD + ANPE 250 mg/kg/day, G_4_; Hypercholesterolemia+ SBD+ ANPE 500 mg/kg/day, G_5_; Hypercholesterolemia+ SBD+ ANPE 750 mg/kg/day; NS, not significant; **, p<0.01.**Supplementary Table 4: Histopathological scores of liver tissues in control and treated groups**

| **Group** | **Treatment** | **Hepatocyte Arrangement** | **Central Vein Congestion** | **Sinusoidal Dilation** | **Inflammation** | **Steatosis** |
| --- | --- | --- | --- | --- | --- | --- |
| G_1_ | Control | 0.12±0.04 | 0.10±0.03 | 0.08±0.02 | 0.11±0.04 | 0.09±0.02 |
| G_2_ | Diseased | 2.18±0.20 | 2.22±0.24 | 2.15±0.19 | 2.10±0.18 | 2.12±0.21 |
| G_3_ | Treated 1 | 1.15±0.14 | 1.18±0.16 | 1.18±0.13 | 1.12±0.12 | 1.08±0.11 |
| G_4_ | Treated 2 | 1.20±0.15 | 1.14±0.13 | 1.10±0.14 | 1.16±0.12 | 1.15±0.10 |
| G_5_ | Treated 3 | 0.10±0.04 | 0.12±0.06 | 0.09±0.03 | 0.11±0.05 | 0.10±0.04 |

Means having similar alphabets do not differ significantly (p-values > 0.05), ANOVA test followed by the post-hoc Tukey’s HSD test, G_1_; SBD, G_2_; Hypercholesterolemia + SBD, G_3_; Hypercholesterolemia + SBD + ANPE 250 mg/kg/day, G_4_; Hypercholesterolemia+ SBD+ ANPE 500 mg/kg/day, G_5_; Hypercholesterolemia+ SBD+ ANPE 750 mg/kg/day.

**Supplementary Table 5: Histopathological scores of kidney tissues in control and treated groups**

| **Group** | **Treatment** | **Glomerular Damage** | **Tubular Degeneration** | **Interstitial Inflammation** | **Cast Formation** |
| --- | --- | --- | --- | --- | --- |
| G_1_ | Control | 0.10±0.06 | 0.12±0.08 | 0.09±0.07 | 0.05±0.02 |
| G_2_ | Diseased | 2.15±0.18 | 2.20±0.22 | 2.10±0.16 | 1.12±0.11 |
| G_3_ | Treated 1 | 1.18±0.14 | 1.10±0.12 | 1.08±0.10 | 0.19±0.08 |
| G_4_ | Treated 2 | 1.22±0.16 | 1.04±0.12 | 1.15±0.13 | 0.14±0.07 |
| G_5_ | Treated 3 | 0.15±0.09 | 0.63±0.10 | 1.02±0.09 | 0.10±0.05 |

Means having similar alphabets do not differ significantly (p-values > 0.05), ANOVA test followed by the post-hoc Tukey’s HSD test, G_1_; SBD, G_2_; Hypercholesterolemia + SBD, G_3_; Hypercholesterolemia + SBD + ANPE 250 mg/kg/day, G_4_; Hypercholesterolemia+ SBD+ ANPE 500 mg/kg/day, G_5_; Hypercholesterolemia+ SBD+ ANPE 750 mg/kg/day.

**Supplementary Table 6: Docking score of the selected compounds against receptor protein oxidosqualene cyclase**

| **Compound Name** | **Docking Score** |
| --- | --- |
| Gallic acid | -5.9 |
| Catechin | -7 |
| Epigallocatechin gallate (EGCG) | -6.8 |
| Ellagic acid | -7.5 |
| Quercetin | -7.6 |
| β-Amyrin | -8.7 |
| Betulin | -7.9 |
| β-Sitosterol | -9.7 |
| Kaempferol | -7.2 |
| Chlorogenic Acid | -6.7 |
